# Seroprevalence, Associated Factors, and Fetomaternal Outcome in Pregnant Women That Tested Positive to Hepatitis E Antibodies in Nigeria

**DOI:** 10.1155/2021/9341974

**Published:** 2021-10-21

**Authors:** Valentine Chimezie Okwara, Ikechukwu Innocent Mbachu, Victor Ikechukwu Ndububa, Henry Chima Okpara, Chioma Pauline Mbachu

**Affiliations:** ^1^Department of Obstetrics and Gynaecology, Imo State University Teaching Hospital, Orlu, Imo State, Nigeria; ^2^Department of Obstetrics and Gynaecology, Nnamdi Azikiwe University, Awka, Anambra State, Nigeria; ^3^Department of Chemical Pathology, Nnamdi Azikiwe University, Awka, Anambra State, Nigeria; ^4^Department of Paediatrics, Nnamdi Azikiwe University, Awka, Anambra State, Nigeria

## Abstract

**Background:**

Hepatitis E virus infection is an emerging disease with varied courses in pregnancy. There is a dearth of statistics among pregnant women.

**Aim:**

To evaluate the prevalence, associated factors, and pregnancy outcome in women that tested positive for hepatitis E virus (HEV) antibodies in pregnancy. *Research Methods*. This was a cross-sectional study conducted among pregnant women at a teaching hospital in Nigeria. Relevant information was collected using a structured questionnaire. Blood was collected from each of the participants, and the serum was used to determine the presence of hepatitis E immunoglobulin M (IgM) and G (IgG). The data were analysed using SPSS version 23. Associations between variables were determined at a *p* value of <0.05.

**Results:**

A total of 200 pregnant women participated in this study. The prevalence of HEV infection among pregnant women was 28.00% (56/200). The mean age was 30.11 ± 5.88. Hepatitis E infection was significantly associated with age (*p* value = 0.028), method of faecal disposal (*p* value = 0.043), and source of drinking water (*p* value = 0.039). A total of 9/200 (4.50%) stillbirths were recorded with 3/9 (33.33%) in women that tested positive for HEV antibodies. About 4/200(2.00%) miscarriages were recorded, and 2/4 (50.00%) were in women that tested positive for HEV antibodies. Hepatitis E infection was not significantly associated with perinatal outcome (*p* value = 0.45). Only 1/56 (0.50%) maternal death was recorded among women that tested positive to hepatitis E, and none was recorded among those that tested negative to hepatitis E antibodies.

**Conclusion:**

There was a significant statistical association between HEV infection and age, method of faecal disposal, and source of drinking water. This underscores the importance of the provision of clean water and safe faecal disposal. Hepatitis E virus infection did not significantly affect the foetal and maternal outcomes.

## 1. Introduction

Hepatitis E virus (HEV) infection is an emerging disease-causing viral hepatitis with both benign and severe courses which depends on the study population. Globally, it is estimated that 20 million people become infected with HEV every year resulting in about three million acute illnesses and 57,000 deaths annually in developing countries, mainly Africa and Asia [1]. A significant mortality rate of up to 30% and above has been reported among infected pregnant women and 30,000 stillbirths primarily those in their third trimester making the disease a public health burden [1, 2].

It has been documented that the genotype of the HEV plays an important role in the severity of the disease. Four genotypes have been documented in the literature [3, 4] Genotype 1 includes isolates from Asia, the Middle East, and North Africa, while genotype 2 has been found in Mexico and Nigeria. Genotype 3 was recovered from swine in North America, Europe, Egypt, Asia, and New Zealand and from humans in North and South America, Europe, Japan, and China. Genotype 4 was found in humans and swine in Asia. These genotypes are important as they correlate with the severity of infection with genotype 1 responsible for most severe infections.

Several risk factors for HEV infection have been documented. These include poor sanitation, poor disposal of faeces, contamination of water supplies, ingestion of undercooked meat and shellfish, overcrowded temporary camps, and transfusion of infected blood products [5–7]. HEV infection has been established as a zoonosis, but outbreaks have been majorly linked to waterborne sources [8].

The prevalence and course of HEV infection vary depending on certain characteristics of the study population. While the disease usually runs a benign course in the healthy nonpregnant populations, the outcome is variable in immunocompromised people and pregnant women with a maternal mortality of 50% and above documented in some studies [9, 10]. There is an associated increase in preterm deliveries, fulminant hepatitis, and intrauterine foetal death in HEV-positive mothers [11]. Vertical transmissions have also been established, causing serious foetal and neonatal infections with significant foetal loss, poor foetal outcome, stillbirths, and neonatal deaths [3, 4, 11, 12]. Mishra et al. [13] reported high perinatal mortality of 69% and maternal mortality of 54%. Boccia et al. [14] recorded a prevalence rate of 24.1% and a case fatality ratio of 31.1%. However, Alkali et al. [15] reported a prevalence of 9.9% in pregnant women in Sokoto, northern Nigeria.

One of the challenges in the diagnosis of HEV infection in pregnancy is the nonspecific nature of the symptoms which include fever, jaundice, muscle weakness, and vomiting. These are symptoms regularly associated with malaria and other common causes of acute febrile illnesses; thus, it may be overlooked as a major contributor to maternal and foetal complications including death [16, 17]. This may likely occur where there is no documented regional or local data. Teshale et al. [2] observed that 0.6% of jaundice in pregnancy was caused by acute viral hepatitis and that HEV infection accounted for 60% of these cases. This is consistent with other studies in central India and Pakistan that showed that HEV infection accounted for 58% and 62% of cases of jaundice caused by viral hepatitis, respectively [11]. Fulminant hepatic failure was more common and more HEV infected died, had obstetric complications, or had worse foetal outcomes than did women with jaundice and acute viral hepatitis caused by other hepatitis viruses [11]. Pregnant women who were infected with HEV had fewer live births and more preterm births.

Despite the potential of HEV infection-causing foetal and maternal complications, it is understudied in pregnancy especially in developing countries that bear most of the disease burden. There is a dearth of studies on HEV infection among pregnant women in Nigeria. This study evaluated the prevalence, associated factors, and pregnancy outcomes of HEV infection in pregnant women in Nigeria.

## 2. Materials and Methods

The study was a cross-sectional analytic study of pregnant women that presented for antenatal care and delivered at the study hospital. The study was conducted at the antenatal clinic and labour ward of Imo State University Teaching Hospital, Orlu, over a ten-month period.

The inclusion criteria were pregnant women at their second and third trimester irrespective of parity and age who gave consent and signed the informed consent form for the study. Ethical approval was obtained from the institution's ethical board. Nonpregnant women were excluded from the study.

A minimum sample size of 202 was calculated using the formula for cross-sectional studies [18] with standard normal deviation set at 1.96 corresponding to 95% confidence level and level of significance set at 0.05, using 12% proportion of target population in a previous study [19] and an attrition rate of 20%.

A total of 209 participants were recruited for the study. However, only 200 participants that completed the study were used in the analysis. Participants were recruited consecutively until the sample size was achieved.

Informed consent was obtained from all the participants. Sociodemographic data, risk factors, symptoms, and obstetric data were obtained from the participants by use of a structured questionnaire. Participants were clinically examined for the presence of jaundice. The delivery details and maternal and neonatal outcomes were documented after delivery. Four millilitres of venous blood were collected into a plain bottle from each of the participants after explaining the procedure. The blood specimen was centrifuged at 3000 rpm for 5 mins. The supernatant serum was transferred to storage tubes and stored at −20°C before batch analysis using Monocent Inc.'s HEV IgM and IgG ELISA test system (Canoga Park, CA, USA).

Serum from all the participants was tested for seroreactivity to anti-HEV IgM and anti-HEV IgG antibodies according to the manufacturer's instruction. Positive and negative control specimen was run concurrently with the subject's sera as part of quality assurance.

### 2.1. Interpretation of Results

The presence of IgM antibodies indicated a recent HEV infection, while the presence of IgG antibodies was indicative of a previous HEV infection. A negative result indicated that the patient has not been infected by HEV.

### 2.2. Statistical Analysis

Data were analysed using the Statistical Package for Social Sciences (SPSS) version 23 (IBM Corp 2015). Numerical variables like age were summarized using mean and standard deviation. Associations between variables were tested using the chi-square tests. The level of significance for all significant tests was set at 5% (*p* < 0.05).

## 3. Results

**Figure 1:**
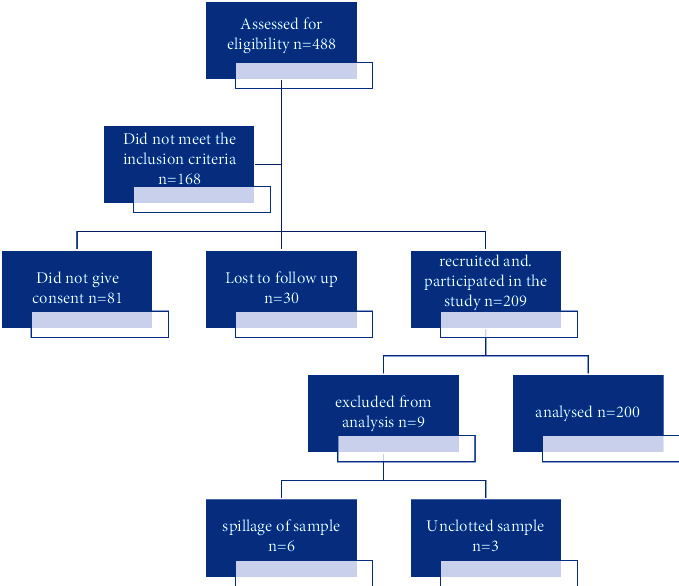
Flow chart of the study.

**Figure 2:**
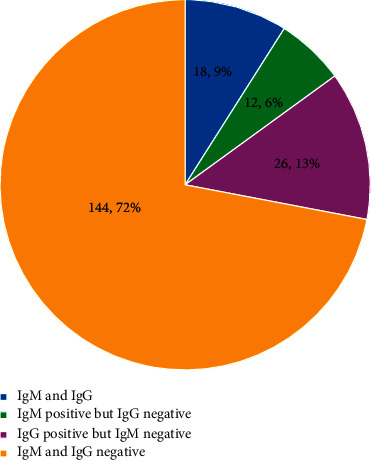
Seroreactivity to hepatitis E IgG and IgM among the pregnant women.

A total of 209 pregnant women were recruited for this study. 200 women completed the study and were included in the final analysis (9 women were excluded from the analysis due to sample spillage and loss to follow-up). This is shown in Figure 1. Of the sera of 200 participants analysed, 56(28.0%) were positive for either HEV IgM and/or IgG giving a seroprevalence rate of 28.0%. Eighteen (9.0%) tested positive for both anti-HEV IgM and IgG antibodies. Twelve (6.0%) tested positive to anti- HEV IgM but negative to HEV IgG antibodies, while 26(13.0%) tested positive to only HEV E IgG antibodies.  Figure 2 shows the seroreactivity to hepatitis E antibodies.

The mean age of participants was 30.11 ± 5.88. The overall age range of participants was 18 to 43 years with the highest population 57(28.50%) within the age range of 30–34 years. The prevalence of HEV infection was significantly higher in the age range of 25–29 years (24/54, 38.89%). There was a significant association between HEV infection and age (*p*=0.028). Most of the participants had tertiary education 114 (57.00%). The seroprevalence of hepatitis E virus was highest in participants with tertiary education 29/114 (20.71%), while the highest percentage was recorded among the participants with secondary education 23/75 (30.67%). There was no significant association between hepatitis E infection and educational status (*p*=0.542). The relationship between sociodemographic profile and hepatitis E infection is shown in Table 1.

Table 2 shows the association between risk factors and hepatitis E infection among the participants. Most participants admitted to washing hands before a meal and after defecation. 55 of the 191 (28.80%) participants who always washed hands before a meal were seropositive, while 32/125 (25.60%) participants who always washed hands after defecation were seropositive. There was no significant association between the presence of HEV antibodies and washing hands before a meal and washing hands after defecation, respectively (*p* value = 0.21 and 0.22). The use of a water closet was the predominant way of faecal disposal, with 159/200 (79.50%) participants using this option. There was a significant association between HEV infection and the method of faecal disposal (*P*=0.043). The commonest sources of drinking water were sachet 95/200 (47.50%) and borehole 93/200 (46.50%). There was a significant association between HEV infection and the source of drinking water (*p*=0.039).

137 participants (68.50%) had at least an episode of diarrhea, and 40/137 (29.20%) of them tested positive for HEV antibodies. Only 76/200 (38.00%) had at least an episode of fever and 20/76 (26.32%) being seropositive to HEV antibodies. Fewer participants 49/200 (24.50%) had a history of blood transfusion with 12/49(24.49%) seropositive for HEV antibodies. Thirty participants had jaundice during their pregnancy with 7/30 (23.33%) of them showing seropositivity. There was no significant association between the symptoms of hepatitis E and HEV antibodies among the study participants. The symptoms of hepatitis E infection are in Table 3.

Women whose parity was between 2 and 4, were majority (96/200 (48.00%)) and had the highest seroprevalence of 26/96 (27.08%). There was no significant association between HEV infection and parity (*p* value = 0.930). Most participants 190/200 (95.00%) had term deliveries 190/200 (95.00%) with 56/190 (29.47%) testing positive to HEV antibodies. There was no significant association between gestational age at delivery and HEV antibodies (*p* value = 0.81). A total of 185/200 participants had a spontaneous vaginal delivery, and 53/185 (28.65%) were seropositive to HEV antibodies. There was no significant association between HEV infection and the route of delivery (*p* value = 0.220). Four of the 200 (2.00%) participants had a spontaneous miscarriage with 2/4 (50.00%) being seropositive to HEV antibodies. A total of 9/200 (4.50%) had stillbirth with 3/9 (33.33%) testing positive to HEV antibodies. The perinatal outcome was not significantly associated with HEV infection (*p* value = 0.45). The only maternal death 1/200(0.5%) recorded in the study tested positive to HEV antibodies (IgM) giving maternal mortality among HEV positive women to be 1/58(1.72%).  Table 4 shows the relationship between HEV infection and the obstetrics variables.

## 4. Discussion

The seroprevalence of 28% of hepatitis E infections recorded in our study is comparable to observation by Delia Boccia et al. [14], who reported a prevalence rate of 24.1% among pregnant women in Darfur Sudan. A much higher prevalence of 45.2% was reported by Sharda Patra et al. [11] in women in India and 84.4% was reported from Egypt, respectively [4]. However, Alkali et al. [15] reported a lower prevalence rate of 9.9% among pregnant women in a mixed study involving pregnant and nonpregnant women in Sokoto, Nigeria. This discrepancy in HEV infection seroprevalence may be linked to rural-urban difference, socioeconomic status, cultural, sanitary conditions, study design, and whether it is sporadic or outbreak in nature.

In the current study, HEV seroprevalence increased with age. This is in accordance with previous reports by Tadesse et al. [20] and Adesina et al. [21] which showed that seroprevalence of HEV infection increases with age. The educational qualification did not have any significant association with HEV infection. This contrasted with the findings by Stroszek et al. [4] who reported increasing seroprevalence with low educational qualification, possibly from lack of knowledge about avoidable risk factors associated with HEV infection. However, this discrepancy from the current study may be due to the high proportion of women who had tertiary education with fewer participants with low educational status.

The current study observed a significant association between HEV antibodies and the source of drinking water. Similar observations have been reported in previous studies. Stroszek et al. [4] noted that exposure to HEV is generally increased in areas with poor sanitation and faecal contamination of their water supply. Hilary et al. [22] observed that faecal contamination of water and food was associated with HEV infection of 10.8% and 65.7% in humans and animals, respectively. Busson et al. [23] reported that poor sanitation and food sources, contaminated water supplies, or uncooked shellfish exposed residents of the Niger Delta region of Nigeria to HEV sporadic cases throughout the year.

The practise of hand washing before food and after defecation was not significantly associated with HEV infection. This is like the study by Eker et al. [24] which also showed no relationship between hand washing and HEV. This could be explained by the observation that most of the participants wash their hands before eating and after defecation.

The current study reported no significant association between HEV infection and a history of blood transfusion. This agrees with the findings of Bello et al. [25]. However, emerging evidence has shown that that HEV infection is an emerging potentially new threat to blood transfusion and safety, after several cases of transfusion transmission were reported [26, 27].

The study did not observe any significant association between HEV infection with fever. This is not surprising because the study was conducted in a tropical environment where malaria infection is endemic. These symptoms are regularly associated with malaria and other common causes of acute febrile illnesses and are well recognized as a major contributor to maternal and foetal complications including death [16, 17]. Maternal jaundice was also not significantly associated with HEV infection. This could be related to a few number of cases of jaundice in pregnancy observed in the study and the presence of other ailments that may present with jaundice in pregnancy. The few number of jaundice cases recorded in this study may also be related to the degree of the severity of the disease.

In the current study, there was only one maternal death among women with HEV infection (1.72%). This death was due to eclampsia. It is difficult to determine if it was related to HEV infection as autopsy was not performed. We did not record any case of fulminant hepatitis; thus, this could be responsible for the low mortality rate recorded in this study. Other studies with a significant rate of fulminant hepatitis recorded high maternal mortality [28, 29]. The reason for the low level of fulminant hepatitis is not clear. It may be explained by the type of genotype predominant in the study area. More so, the immune status of the participants may have played a significant role in reducing the severity of the disease.

The perinatal outcome was not significantly associated with HEV infection. This is contrary to some previous studies which observed that HEV infection was associated with increased rates of spontaneous abortion, intrauterine foetal death, and preterm labour which has been attributed to the high rate of vertical transmission seen in HEV [11, 16]. This may be linked to the severity of maternal disease which is influenced by the genotype of the hepatitis E virus common in each locality, the immune status, and the rate of vertical transmission.

Previous studies of HEV infection among pregnant women in Nigeria have focused mainly on the prevalence of hepatitis E. The index study is one of the few studies in Nigeria that determined the pregnancy outcome in women with HEV infection. This study may serve as an impetus and a foundation for more studies in Nigeria.

Despite these obvious strengths, this study has some limitations. The study was conducted in a public tertiary hospital; thus, it should be generalised with caution.

## 5. Conclusion and Recommendation

In conclusion, there is a high burden of hepatitis E infection among pregnant women which has a significant association with age, method of faecal disposal, and source of drinking water. In the absence of curative treatment, key preventive strategies should include public enlightenment on the risk factors, provision of clean portable pipe-borne water, and proper and adequate environmental sanitation.

We infer that maternal and perinatal outcome in women with nonsevere HEV infection is low.

## Figures and Tables

**Table 1:** Relationship between sociodemographic profile of participants and hepatitis E infection.

Parameter	Frequency	Hepatitis E infection		Total	*p*-value
		Negative	Positive		
*Age range*					**0.033**
<20	4	4	0	4	
20–24	35	31	4	35	
25–29	54	33	21	54	
30–34	56	42	14	56	
≥35	51	34	17	50	

*Highest educational attainment*					0.542
Primary	11	7	4	11	
Secondary	75	52	23	75	
Tertiary	114	85	29	114	

**Table 2:** Risk factors and hepatitis E infection among the participants.

Parameter	Frequency	Hepatitis E infection		*p*-value
		Negative	Positive	
*Washing of hand before meal*				0.22
Always	191	136	55	
Sometimes	9	8	1	

*Washing hand after defecation*				0.21
Always	125	93	32	
Sometimes	75	51	24	
*Method of faeces disposal*				**0.043**
Water closet	159	113	46	
Pit	39	31	8	
Bush	2	0	2	

*How do you dispose house refuse*				0.88
Burning	27	19	0	
Bush	30	21	9	
Pit	20	16	4	
Evacuation site	123	88	35	

*Source of drinking water*				**0.039**
Sachet	95	66	29	
Borehole	93	72	21	
Bottled water	4	3	1	
Rain	7	2	5	

*No of people per room*				0.81
1	8	6	2	
2	77	53	24	
3	79	58	21	
4	30	23	7	
5	6	4	2	

**Table 3:** Symptoms of hepatis E Infection among the study participants.

Parameter	Frequency	Hepatitis E infection		*p*-value
		Negative	Positive	
*History of diarrhea*				0.58
Yes	137	97	40	
No	63	47	16	

*History of fever during pregnancy*				0.19
NO	120	84	36	
1 episode	56	45	11	
>1 episode	24	15	9	

*Past history of blood transfusion*				0.33
No	151	107	44	
Yes	49	37	12	

*History of yellowness of the eyes*				0.54
No	170	121	49	
Yes	30	23	7	

*History of yellowness of eyes in the family*				0.09
No	187	132	55	
Yes	13	12	1	

**Table 4:** Obstetric variable and hepatitis E infection.

Parameter	Frequency	Hepatitis E infection		*p*-value
		Negative	Positive	
*Parity*				0.93
1	77	55	22	
2–4	96	70	26	
>4	27	19	8	

*GA at delivery*				0.81
<28	5	3	2	
28–33	1	1	0	
34–36	1	1	0	
37–42	190	141	56	
>42	3			

*Route of delivery*				0.22
SVD	185	132	53	
CS	11	10	1	
Miscarriage	4	2	2	

*Fetal/neonatal outcome*				0.45
Alive birth	187	136	51	
Stillbirth	9	6	3	
Miscarriage	4	2	2	

*Maternal outcome*				
Alive	199	144	55	
Death	1	0	1	

## Data Availability

The data are available from the corresponding author upon reasonable request.
